# Polynomial-Time Algorithm for Controllability Test of a Class of Boolean Biological Networks

**DOI:** 10.1155/2010/210685

**Published:** 2010-07-08

**Authors:** Koichi Kobayashi, Jun-Ichi Imura, Kunihiko Hiraishi

**Affiliations:** 1 School of Information Science, Japan Advanced Institute of Science and Technology, Nomi, Ishikawa 923-1292, Japan; 2 Graduate School of Information Science and Engineering, Tokyo Institute of Technology, Oh-okayama, Tokyo 152-8552, Japan

## Abstract

In recent years, Boolean-network-model-based approaches to dynamical analysis of complex biological networks such as gene regulatory networks have been extensively studied. One of the fundamental problems in control theory of such networks is the problem of determining whether a given substance quantity can be arbitrarily controlled by operating the other substance quantities, which we call the controllability problem. This paper proposes a polynomial-time algorithm for solving this problem. Although the algorithm is based on a sufficient condition for controllability, it is easily computable for a wider class of large-scale biological networks compared with the existing approaches. A key to this success in our approach is to give up computing Boolean operations in a rigorous way and to exploit an adjacency matrix of a directed graph induced by a Boolean network. By applying the proposed approach to a neurotransmitter signaling pathway, it is shown that it is effective.

## 1. Introduction

Various approaches to modeling, analysis, and control synthesis of biological networks such as gene regulatory networks and metabolic networks have been recently developed in the control community as well as the theoretical biology community [1]. In these approaches, it is one of the final goals to develop systematic drug discovery and cancer treatment [2, 3]. Biological networks in general can be expressed by ordinary/partial differential equations with high nonlinearity and high dimensionality. Since such complexities cause difficulties in analysis and control design, various simpler models such as Petri nets, Bayesian networks, Boolean networks, and hybrid systems have been proposed for dealing with complex and large-scale biological networks at the expense of rigorous analysis (see e.g., [4, 5]).

This paper discusses the controllability problem of biological networks. In gene regulatory networks, for example, the controllability problem is defined as the problem of determining whether expressions of genes of interest can be arbitrarily controlled by expressions of a specified set of the other genes. As far as we know, two approaches to the controllability analysis of such biological networks have been developed so far: a piecewise affine model-based approach and a Boolean network model-based approach. However, the former approach can be applied to only the class of relatively low-dimensional systems [6, 7].

On the other hand, a Boolean network model, where binary state variables are assigned to nodes and the transition rules of the state are given by Boolean functions [8, 9], will be more practical for analysis of *large-scale* biological networks thanks to its bold simplification. Akutsu et al. have recently discussed the controllability problem of Boolean networks with control nodes and controlled nodes and have proven that this problem is NP-hard in a general setting [10]. Furthermore, they have proposed a polynomial-time algorithm for the classes of networks including a tree structure or at most one loop, and an exponential-time algorithm for the other classes. Indeed there is a criticism that a Boolean network model is too simple as a model of biological networks, but for large-scale networks it will be able to provide some indication or clue towards further detailed analysis. Thus various approaches based on this model have been well-studied so far (see e.g., [11–19]).

Motivated by the theoretical results in [10], this paper also focuses on the controllability problem of Boolean networks with control nodes and controlled nodes and proposes a sufficient condition for the Boolean network to be controllable, which can be easily verified by a polynomial-time algorithm. Our standing point is to give up computing complex Boolean operations in a rigorous way and to focus on deriving an easily-checkable sufficient condition for controllability so as to be applied to large-scale networks. The obtained algorithm is based on simple operations on an adjacency matrix of a directed graph induced by a Boolean network. This is a remarkable point of our approach, different from the method in [10], and enables us to apply our approach to a wider class of Boolean networks including nontree structures.

First, after the definition of controllability of Boolean network models with control nodes and controlled nodes is described, a sufficient condition for the controllability is derived in the form of an algorithm. Next, the computational complexity for the algorithm is discussed to show that it is a polynomial-time algorithm. In addition, PC-based numerical experiments show that the obtained algorithm is applicable to a class of Boolean networks with at least 1000 nodes. Finally, as an illustrative example, the proposed algorithm is applied to the Boolean network model of a neurotransmitter signaling pathway [20], which expresses an interaction pathway between the glutamatergic and dopaminergic receptors. Note that the polynomial-time algorithm proposed in [10] cannot be always applied to this problem. This Boolean network model consists of 16 nodes, and the problem of simultaneously controlling two important nodes among them, that is, concentration of exocytosis and phospholipase C, is discussed based on the proposed algorithm. As a result, we show that for example, they can be simultaneously controlled by keeping substance concentration at the other 4 nodes constant with appropriate values.

*Notation 1.* Let  denote the set of nonnegative integers and  the set of  matrices consisting of elements  and . We also denote by  and  the  identity matrix and the  zero matrix, respectively. For simplicity of notation, we sometimes use the symbol  instead of  and the symbol  instead of . Let  express the transpose of the matrix .

## 2. Boolean Network Models

This section provides a brief review on a Boolean network model [8, 9]. A Boolean network model consists of a set of nodes and a set of regulation rules for nodes, where each node expresses a gene, a molecule, or an event in the genetic network. The state variable  at node  takes a Boolean value of  or  representing "inactive" or "active" status of the node, respectively. A regulation rule for each node is given in terms of a Boolean function, and each node state changes synchronously.

**Figure 1:**
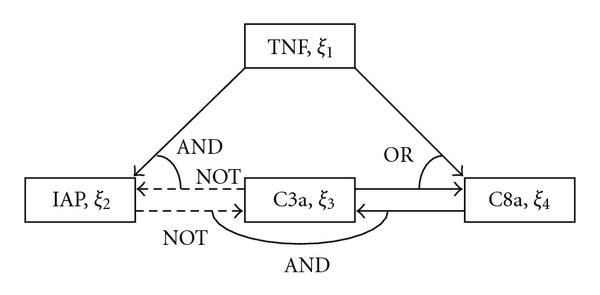
**Simplified model of an apoptosis network**. Activation (solid), Inhibition (broken).

As an example, we consider a very simple and interesting Boolean network model of an apoptosis network in Figure 1 given by (1)

where , , and  denote logical NOT, AND, and OR, respectively,  denotes the discrete time, the concentration level (high or low) of the tumor necrosis factor (TNF, a stimulus) is denoted by , the concentration level of the inhibitor of apoptosis proteins (IAP) by , the concentration level of the active caspase 3 (C3a) by , and the concentration level of the active caspase 8 (C8a) by . Here if the binary variable  has the value of "1", then the concentration of a certain reactant gets larger than a prescribed threshold (i.e., it is active), otherwise less than that. In addition, logical NOT corresponds to inhibition of gene expressions.

Since the caspase C3a is responsible for cleaving or breaking many other proteins, a high-level of the C3a concentration, that is,  implies cell near-death; otherwise, cell survival. As seen in (1), if the concentration of IAP is high () or the concentration of the caspase C8a is low (), then the concentration of C3a gets low, that is, . On the other hand,  and  at the next time depend on the value of  as well as . In this way, some dynamical interactions exist. See [21, 22] for further details.

A general form of a Boolean network model is given by the state equation (2)

where  is the state vector at time , and  is a Boolean function, where logical operators consist of AND (), OR (), NOT (), and XOR ().

## 3. Problem Formulation

In a Boolean network model (2), the state  is uniquely determined by giving the initial state , which implies that (2) is an autonomous system and has no control nodes.

On the other hand, this paper will consider the Boolean network model with control (i.e., input) nodes and controlled (i.e., output) nodes to discuss the output-controllability of this model. This model is given by (3)

where each element of  denotes the state of the control node whose value can be arbitrarily given as an external control input in the Boolean network, each element of  denotes the state of the node except for the control nodes in the Boolean network, and each element of  denotes the state of the node to be controlled as an output in the network. Note here that  does not imply a measured output. Hereafter according to control theory, , , and  are called a "state", "control input" and "output", respectively. In addition,  is a Boolean function, and  is the output matrix satisfying for each element  of (4)

Furthermore, the product of  and  in  expresses a product operation on matrices/vectors of the real number field. Thus the above condition on  guarantees that the output is the state variable itself, that is, for each  there exists  such that  holds. The case of  is also included here. This condition on  will not be restrictive in analyzing controllability of biological networks such as gene regulatory networks, since the relation on regulation among genes/molecules will be mainly discussed there.

For the system  of (3), the notion of output-controllability is defined as follows.

*Definition 1.* Suppose that for the system  of (3), the finite time  and the initial state  are given. Then the system  is said to be *-output-controllable* at  if for every , there exists a control input sequence , , such that . Furthermore, the system  is said to be *-output-controllable* if it is *-output-controllable* at every .

The above notion of controllability comes from the fact that, for example, in control of genetic networks we often would like to determine if expressions of certain gene of interest (corresponding to ) will be able to be inhibited (or activated) by means of appropriately adjusting the expressions of a given set of genes (corresponding to ). It is remarked that we assume that the control time  is explicitly specified in the above definition.

Let us get back to the Boolean network model (1) of an apoptosis network. As discussed in [21, 22], we consider (TNF) itself as a control input. So by ignoring the dynamics on , that is, , we suppose in (1) that  and , which yields (3) of the form (5)

where  denotes the -th element of . As for the output , either case of (6)

can be treated by assumption. Then let us verify the -output controllability of the system (5). As discussed in Section 2,  expresses cell near-death, and  expresses cell survival. So we would like to know if the system is -output-controllable with respect to the output . Suppose that  (i.e., the initial states of IAP, C3a and C8a are all low-level),  (i.e., ), and . Then since  holds independently of  by simple calculation, we see that system (5) is not -output-controllable at , which implies that we cannot control the system from the state "cell survival" within 2 time steps no matter how the control value of  is given.

On the other hand, suppose in (1) that  and . Then we obtain (3) of the form (7)

where  is ignored. Suppose that , , and (8)

(i.e., ). Then since  and  are obtained, we see that system (5) is -output-controllable at , for example, (a)  for , , (b)  for , , (c)  for , , and (d)  for , . This implies we can *simultaneously* control the value of  and  at . In this way, the proposed controllability enables us to verify the existence of a control input sequence such that the output has the desired value in a given finite time, and the obtained result indicates how to give the value of a control input sequence.

Next, we will explain our basic strategy for deriving the controllability condition. Let us consider a Boolean network expressed as the state equation (9)

which is given by [10]. Although this model is very simple, it provides significant clues to address this problem. For the Boolean network model (9), we can consider three possible specifications, choosing either , , or  to be the control input for the system.

First, suppose that  and , that is,  itself is the control input. Then it follows that (10)

Note here that  is ignored because we assume that  itself is the control input. As for the output , either case of , ,  can be considered in this case. Consider the controllability of the system (10) with  (i.e., ) for . In this example, we will consider whether system (10) is -output-controllable or not by directly calculating state trajectories of each system. From (10), we have (11)

So if ,  holds irrespective of the value of , similarly for the case of . Therefore, we see that system (10) is not -output-controllable. In the same way, we see that system (10) is not -output-controllable in every case of , , and  for .

Secondly, suppose that  and , that is,  itself is regarded as the control input. Then we obtain (12)

where  is ignored. Consider the controllability of the system (12) for . From (12) we have (13)

Thus we see that the system is not -output-controllable for , while that the system is -output-controllable with  for both cases of  and .

Finally, suppose that  and . Then we obtain (14)

where  is ignored. Consider the system (14) with . From (14), we have (15)

which implies that system (14) is -output-controllable. However, in the case of , we have (16)

which implies that the system (14) is not -output-controllable.

Note that for (11) with , we see that the controllability property does not hold due to the fact that  directly depends on . On the other hand, for (13) with ,  is adjacent to  and  in the Boolean network, which implies that  is arbitrarily given by  and . In a similar way,  is adjacent to . However,  cannot be realized by  and  because  always holds when . These examples are very important in discussing the controllability in a Boolean network, that is, if the Boolean function of  includes an initial state , or includes the same input in the outputs at the same time, then the system in question is not -output-controllable. In the following section, by motivating the above discussion, we will consider to derive a controllability condition.

*Remark 1.* In the above example, we assume that when some genes are identified as control inputs, the original dynamics of the corresponding genes can be ignored. However, in the case that the corresponding gene has a strong interaction with other genes, this assumption may not be suitable. One of methods for coping with such a case is to add a new gene (node) that works as the control input [10], where it is called an *external control node*. Our approach below can be also applied to this case.

## 4. Output-Controllability Condition

### 4.1. Preliminaries

This section presents a sufficient condition for the system (3) to be -output-controllable in the form of an algorithm.

Consider a simple example given by (17)

This system has the following relation: (18)

Similarly, we see that , , hold identically. In Boolean functions, identical equations are in general given by (19)

where  is any Boolean function of a vector of binary variables. Obviously such identities on  or  affect the controllability in a Boolean network (note that even if ,  holds irrespective of ).

Let us consider again the Boolean network model (5) of an apoptosis network. If we suppose that ,  (i.e., ), and , then by a simple calculation, we obtain the following identity: (20)

So in Boolean biological networks, there exists the case that identities are appeared. However, identities may not be appeared in the real biological relevance. The reasons why such identities are appeared are that the state is binarized and that a time-delay of the state is ignored. To overcome the latter point, a temporal Boolean network model  has been proposed in [23]. However, identities may appear even in a temporal Boolean network. The output-controllability condition proposed below can be similarly applied to a temporal Boolean network model.

Thus first of all, we will focus on finding such identities in  before discussing a kind of initial condition and a kind of input-independency. This will require the introduction for several symbols.

The following assumption is made.

*Assumption 1.* The Boolean function  in (3) has no redundant variables.

For example, in the logical function ,  holds. So  is a redundant variable, and  can be rewritten as . Any given Boolean function can be changed so as to satisfy Assumption 1: after it is transformed into an appropriate canonical form (e.g., Reed-Muller canonical form (polynomials over the finite field )), it is easy to eliminate redundant variables by expanding based on four operations over . Also in the identification of Boolean network models (e.g., see [24]), since the correlations between variables are checked, the Boolean function  in (3) will satisfy Assumption 1 in many cases. By Assumption 1, it is guaranteed that the Boolean function  itself does not include any identities, although  may include some identities. Let  denote the number of the logical NOT appeared in (3), where the logical NOT operators are distinguished when the corresponding terms are different even if the corresponding variables are the same. In addition, consider the fictitious inputs , , which have one-to-one correspondence with the variables operated by the logical NOT, that is,  or  in (3). Then the system (3) can be equivalently rewritten as the following system: (21)

where the Boolean function  does not include the logical NOT, and (22)

For example, system (17) is rewritten as (23)

subject to .

**Figure 2:**
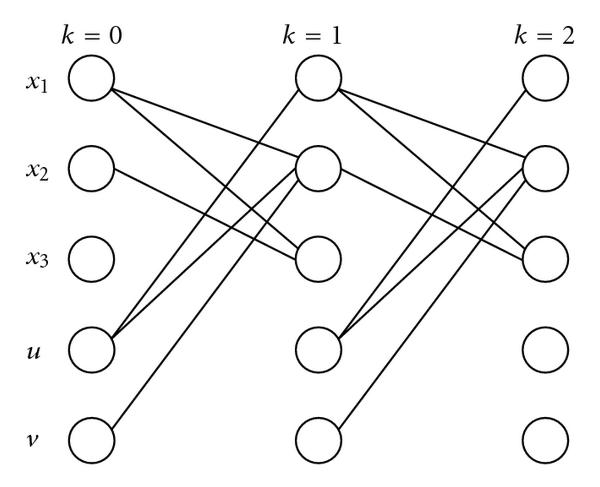
**Temporal/spatial network of the system (23)**.

Next, consider the adjacency matrix  for the directed graph induced by the Boolean network of the system (21). For example, the adjacency matrix for the system (23) is given by (24)

where if there exists an arc from node  to node , then the -th element of  is . Hereafter, without loss of generality, the -th element of  is assigned to node  in the directed graph, where . In the case of (24), , , , , and  are assigned to nodes , , , , and , respectively. Then in Figure 2, which shows a temporal/spatial network of the system (17), we say that for example, there exists a path between  and .

Using the adjacency matrix , we also compute the matrix , , where (25)

In the case of the system (17), we have(26)

where .

For the system (21),  expresses whether there exist paths between  and ,  and , or  and  for any given . In the case of (26), we see that  is adjacent to  and . In other words,  expresses which elements of , , and  are variables of a Boolean function representing . However, note here that from , we cannot specify an explicit form of the Boolean function in question.

Furthermore, the following symbol is used: (27)

where , , and . Let also , , and  denote each element of , , , respectively. If  holds, then there exist  paths between  and . For the state , , of the system (3), let  express the index set of elements of  operated by the logical NOT as . In a similar way, for the control input , , of the system (3), let  express the index set of elements of  operated by the logical NOT as . Here,  holds. In addition, there is a one-to-one correspondence between each element of ,  and the index  of . Let  and  express the index  of  corresponding to  and , respectively. In the case of the system (23), ,  hold, and for ,  holds.

Finally, we define the following matrices: (28)

where (29)

### 4.2. Proposed Algorithm

Now we are in a position to propose a -output-controllability test algorithm. Since this kind of problem is NP-hard [10], we pay our attention on deriving a sufficient condition for the controllability. Although this sufficient condition is given in the form of an algorithm, it is somewhat complex. Thus before describing an algorithm, we describe the outline of the algorithm.

First, we consider a necessary condition for  to include identical equations. From Figure 2 of the example (23), we see that  in (18), which has no identities, has two paths from , and that  is connected to some node on the paths. In this way, if some identical equation exists in , there always exist more than 2 paths from  to some state and also the logical-NOT operations exist on the paths, which is a necessary condition and not necessarily a sufficient condition. Since it will spend huge time to rigorously specify the existence of identities for a large network, we consider here to exclude the cases satisfying the above necessary condition, that is, we do not determine here the controllability in such cases.

Next, for the system that includes no identical equations, we use a kind of input-independency to determine the controllability. For example, consider the case that neither identity on  nor  exists in  and that  is expressed by (30)

as a result of recursive calculation (see Section 6 for such an example), where ,  are some Boolean functions. This system is obviously -output controllable because each  is expressed by different  and no  exists in . From the viewpoint of adjacency relation, this implies that there exists no path between  and , there exists at least one path from each  to some , and each  has a path with only one  or has no path to any . This can be easily found from the adjacency matrix, although it is a sufficient condition for the controllability. This is a rough story of our approach.

The proposed algorithm is given as follows.

*Algorithm 1* (*T*-output-controllability test algorithm). 

  

Part A: Check of the Existence of Identical Equations.

*Step 1.* Set . Compute , , and .

*Step 2.* If , go to Step 6. Otherwise set . Compute , , and .

*Step 3. * If there exists  such that  or  such that , denote them by  or , respectively, and go to Step 4. Otherwise, go to Step 2 if  and go to Step 6 if .

*Step 4. * If there exists  such that  or  such that , and 1 or 1 holds, go to Step 8. Otherwise, go to Step 5.

Step 5.

*Substep 5.1.* Set .

*Substep 5.2.* If any element of -th column or -th column in  is greater than or equal to , go to Step 8. Otherwise, go to Substep 5.3.

*Substep 5.3.* If , set  and go to Substep 5.2, or else go to Step 2.

Part B: Check of the Independence of Each .

*Step 6.* If the following conditions hold for the matrices  and  in (28), system (3) is -output-controllable, or else if only condition (i) does not hold, then go to Step 7. Otherwise go to Step 8.

(i)  holds;

(ii) each column vector of  is a nonzero vector;

(iii) each row vector of  is a zero vector, or has only one element with a nonzero value.

*Step 7.* Suppose  for a given constant vector . Let  denote the index set of elements of  that are constant for any  (). Then if the following condition holds, system (3) is -output-controllable at . Otherwise, go to Step 8.

(iv) For , there exists no  satisfying .

*Step 8.* This algorithm cannot determine whether the system (3) is -output-controllable or not (at ).

The above algorithm allows us to determine the -output-controllability of the system as follows.

First, noting that the identical equations have the form in (19), and  is obtained recursively from (21), we see that the identical equations appeared in  always have the form (31)(32)

where  denotes either variable of  or , (33)

and ,  are some subsets of the index set . Then using the forms of (31) and (32), the following lemma on Part A of Algorithm 1 is obtained. 

**Lemma 1.***In Step 6,  includes neither identities of (31) nor identities of (32).*

*Proof.* In Step 3, from  for some , , and , we see that more than 2 paths from  to  exist, which is necessary for the identity on  to exist (similarly for the case of ). Thus we next focus on the existence of logical NOT (i.e., ) in these paths in Step 4 and Step 5.

Consider the case that the logical NOT (i.e., ) corresponding to  or  obtained in Step 3 exists in (21), in other words, either  or  holds. Then the condition  implies that the term  is included in the paths in question, which is a necessary condition for the existence of the identity in . Thus we exclude this case (Step 4). (similarly for the case ).

In the other case, from (31), (32), for , some , to exist in the paths in question is necessary for the existence of identities. If any element of the -column or the -column of  is greater than or equal to 1, some element of  exists in the paths in question. Thus we exclude this case (Substep 5.2). Therefore, it follows that  includes no identities in Step 6.

From Lemma 1, we see that the case that  includes the identities that have the form of (31) or (32) is excluded from the viewpoint of a necessary condition for the identity to exist in . Thus we obtain the following theorem.

**Theorem 1.***For a given , the following statements hold. *

(i) *the system (3) is -output-controllable if conditions (i), (ii), and (iii) in Step 6 hold subject to Part A of Algorithm 1,*

(ii) *for a given , the system (3) is -output-controllable at  if condition (iv) in Step 7 holds subject to Part A and Step 6.*

*Proof.* First, the statement (i) is proven for the system satisfying the condition that  includes neither identities of (31) nor identities of (32). From Lemma 1, this condition is satisfied in Step 6. Then condition (i) in Step 6 implies that there exists no path between each element of  and each element of , since the -th element of  expresses if a path from  to  exists or not. On the other hand, note that -th element of  expresses if a path from  to  exists or not . Thus condition (ii) in Step 6 implies that there exists at least one path from each element of  to some .

Furthermore, condition (iii) in Step 6 means that the input  for each  and  has a path connected to only one element of  or has no path to any element of . From these conditions, it follows that each  affects at most one  and not the other , . Hence the value of  can be independently specified by the corresponding , which implies that system (21) is -output-controllable.

Next, the statement (ii) is proven. Since condition (i) in Step 6 does not hold, in this case, there exists a path between some element of  and some element of . On the other hand, condition (iv) in Step 7 guarantees that there exists no path between constant elements of  and elements of . Thus  is not affected by the value of . Therefore, from (ii)–(iv), it follows that system (21) is -output-controllable at . This completes the proof.

As an example, consider system (17) again. Suppose . The matrices , ,  of Step 1 are given by (26), and , ,  of Step 2 are (34)

In Step 3, from , we obtain  and . In Step 4, from  and , we have  and . So go to Step 8, that is, it is impossible to determine if system (17) is -output-controllable. In fact, from (18),  includes the identity . Thus we see that there exists an identical equation.

Let us also consider the case of ,  in the system (17). Then for , we have (35)

From Step 1 Step 2 Step 3 Step 6, we can see that the system (17) is -output-controllable. In fact, by simple calculation, the Boolean function of  is derived as .

As for identical equations, the proposed algorithm excludes the case of  as well as (19). This is a weak point of this algorithm. Furthermore, consider the following system: (36)

This system is -output-controllable for . However, the proposed algorithm cannot determine whether this system is -output-controllable or not; thus there exists a class of systems such that the proposed algorithm cannot determine the controllability. Needless to say, it will not be so easy to cope with various cases stated above due to high nonlinearity of Boolean functions.

While the proposed algorithm includes such disadvantages, one of the main advantages of the algorithm is that the computational complexity of the above algorithm is very small. This will be discussed in the following section.

## 5. Computational Complexity Analysis

In this section, we discuss the computational complexity of the algorithm proposed in the previous section.

First, let us recall the definition of the symbols used here. The number of the state, the control input, and the output in (3) are denoted by , , and , respectively. The number of the logical NOT appeared in (3) is expressed by . In addition,  expresses the control time. Then the following result is obtained.

**Lemma 2.***The computational complexity of the proposed algorithm is  for ,  .*

*Proof.* The computation of the proposed algorithm consists of (a) checking each condition of Part A, and (b) checking whether conditions (i) to (iv) hold or not.

First, (b) is considered. The computational complexity to compute  is . So the computational complexity to compute  and  is given by both . Further, the computational complexity to compute the product of  and  is . So by simple calculation, the computational complexity of  is obtained as . The computational complexity of generating  is obviously less than the case of . Therefore, the computational complexity to compute  and  is , which also includes the computational complexity to check conditions (i) to (iv) in Steps 6 and 7 for given  and .

Next, (a) is considered. The matrices , ,  are obtained directly from , and the computational complexity of Step 5 is . As a result, since the computational complexity of each checking in Part A is , the computational complexity of Part A is less than .

Therefore, the computational complexity of the proposed algorithm is given by .

From Lemma 2, we see that the proposed algorithm is a polynomial-time algorithm. Furthermore, the computational time for performing the proposed algorithm is evaluated by numerical experiments, where the total computational time in Part B is measured because from the proof of Lemma 2 we see that the computational complexity of Part B is dominant. So the adjacency matrices to be evaluated are generated randomly for each , where ,  are given. The results are shown in Table 1, where MATLAB on the computer with the Intel Core 2 Duo CPU 3.0 GHz and the 2 GB memory is used. In Table 1, the worst computational time implies the worst value among  cases randomly selected for each . From Table 1, we see that the proposed algorithm can be applied to relatively large-scale Boolean network models.

**Table 1:** Computational time of the proposed algorithm ()

		Worst comp. time [sec]
100	50	0.1
200	100	0.5
300	150	1.5
400	200	3.3
500	250	6.3
600	300	10.2
700	350	15.9
800	400	23.1
900	450	32.4
1000	500	43.6

## 6. Application to Neurotransmitter Signaling Pathway

**Figure 3:**
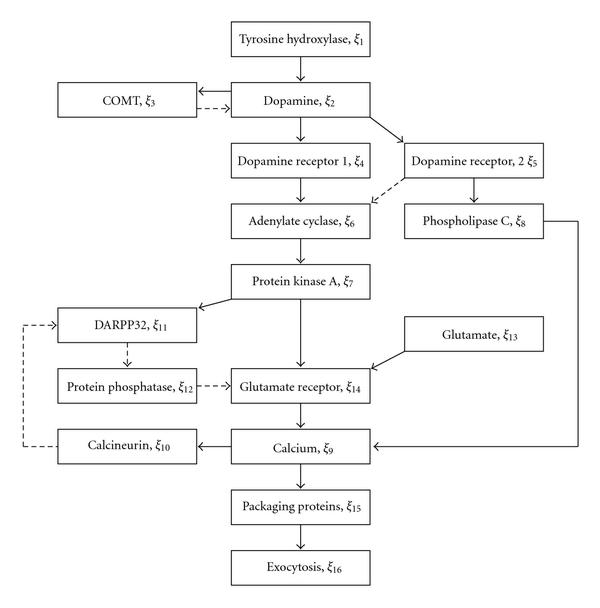
**Simplified model of the interaction pathway between the glutamatergic and dopaminergic receptors**. Activation (solid), Inhibition (broken).

In this section, the proposed algorithm is applied to a Boolean network model of interaction pathway between the glutamatergic and dopaminergic receptors in Figure 3, which has been proposed in [20]. In this pathway, exocytosis, by which a cell directs the contents of secretory vesicles out of the cell membrane, is regulated, depending on the value of neurotransmitters such as dopamine and glutamate. Then it is important from the viewpoint of synaptic plasticity to consider whether exocytosis can be controlled by regulating other elements. In the Boolean network model of Figure 3, the dopamine (neurotransmitter, ) is synthesized by tyrosine hydroxylase () and catabolized by COMT (). The dopamine binds to the dopamine receptor 1 (DRD1, ) and the dopamine receptor 2 (DRD2, ). DRD1 stimulates adenylate cyclase  to activate protein kinase A (), which activates DARPP32 (). DARPP32 inhibits protein phosphatase 1 (). By inhibitation of protein phosphatase 1, activation of protein kinase A, and presence of the glutamate (), the glutamate receptor  is activated to elevate the concentration of the intracellular calcium (). On the other hand, DRD2 inactivates adenylate cyclase and activates phospholipase C () in order to elevate the concentration of the intracellular calcium. The intracellular calcium activates calcineurin (), which inhibits DARPP32. Also, the intracellular calcium activates packaging proteins () and finally exocytosis (). The process of exocytosis of the glutamate receptor expresses one of events in synaptic plasticity, that is, if exocytosis is activated, then the neurotransmitter is secreted out of the cell membrane. In this model, the concentration of the above reactants is expressed by a binary variable , that is,  if it is high, otherwise . Then the state equations of this system are given as (37)

From Figure 3, we see that this Boolean network includes at least four loops, for example, the loop of , , , and , the loop of , , , , and , and so forth. In synaptic plasticity, it is required that the binary value of  expressing exocytosis can be arbitrarily controlled. Furthermore, phospholipase C () is a kind of enzymes that cleaves phospholipids and as a result protein kinase C as well as calcium  are activated. The former, protein kinase C, which works outside of the network in Figure 3, is one of key enzymes in signal transduction pathways. Thus since phospholipase C affects the other significant network, it will be important to simultaneously control the value of  and the value of . Therefore  and  are regarded as the output, that is, . In addition, we assume that  and  cannot be directly controlled.

For a fixed dimension of  and the fixed output , all combinations of , , are considered as the control inputs, which we call the input-combinations. Then for a given , the proposed algorithm is applied to the system of the form (3) obtained for each input-combination of . It is remarked that depending on the choice of the kind of control inputs, there exist several cases to which the polynomial-time algorithm proposed in [10] cannot be applied due to the graph-structure constraints. Furthermore, it is also remarked that even for fixed control inputs, the controllability problem is NP-hard. So the problem of finding efficient control inputs that make the system controllable is further harder than this problem.

By applying our algorithm to the case of each input-combination of  and each fixed , we obtain, for example, the following results. In the case of  and , we can find that among  input-combinations, there exist at least 6 input-combinations of  that make the system -output-controllable. In this way, since the proposed algorithm for each input-combination is very efficient, for example, the computation time via the proposed algorithm is about 10 [sec] for Boolean networks with 600 nodes (see Table 1) and , it enables us to verify the controllability condition for a certain number of input-combinations within a practical time; for example, about 3 [hours] will be required for 1000 input-combinations of a Boolean network with 600 nodes.

In the case of  and , we can also find controllable control inputs among  input-combinations. For example, we obtain as one of combinations of  and  that make the system -output-controllable(38)(39)

It is remarked that the polynomial-time algorithm proposed in [10] cannot be applied to the system with the state (38) and the input (39) because the network includes the two loops, that is, the loop of , , , and , and the loop of , , , and . Furthermore, based on the above result the Boolean function of  can be derived as (40)(41)

which implies that the value of  can be freely given by control inputs, for example, (a)  for , , , , , and (b)  for , , , , .

Finally, we discuss the control input sequence realizing the desired output values. One of criticisms in control of Boolean networks is to assume that the value of the control input can be arbitrarily given at each time. In many biological systems, this assumption is not always satisfied, and input constraints are frequently imposed. One of input constraints is that the value of the control input is given as a constant within a certain sufficiently long time period. Although it is one of future works to explicitly deal with such an input constraint, based on the proposed algorithm, we may also find a constant-valued sequence of control inputs for which the desired values of outputs are obtained. For example, in (40) and (41), let us consider to find a control input sequence satisfying  and . Since , , , , and  are obtained as one of solutions, it is remarked that the following control inputs are given as any binary value: , , , , and , , , , , and , , , , , and , , , , . This allows us to give the values of the control input sequences as a constant, that is, , , , , . Thus the proposed algorithm helps us to find a practically useful control input sequence. Furthermore, this kind of degree of freedom in control inputs may be used for the optimal control problem. Once we can determine control input variables by our algorithm, we can use a tool for finding optimal control input sequences, which have been developed in hybrid control theory, for example, [25, 26]. It is expected that such an analysis will provide one of guidelines in experimental approaches to the control problem of biological networks.

## 7. Conclusion

In this paper, the controllability analysis for biological networks expressed by a Boolean network model with control nodes (inputs) and controlled nodes (outputs) has been discussed. First, a sufficient condition for the Boolean network model to be output-controllable has been derived by exploiting an adjacency matrix of its network graph. The obtained condition, which is given in the form of an algorithm, can be checked in polynomial time with respect to the state/input dimensions and the control time period; it will be one of the powerful tools that can provide some clues for finding effective control inputs to control a large-scale biological network. Next, by PC-based numerical experiments, it has been shown that the proposed method is applicable to large-scale Boolean networks with at least 1000 nodes. Finally, the proposed method has been applied to the Boolean network model expressing a neurotransmitter signaling pathway, and has shown that it is controllable with respect to both exocytosis and phospholipase C when appropriate control inputs are used.

There are many interesting open problems to be addressed in the future. It is one of the most important issues to characterize a class of Boolean networks to which our algorithm can be applied as a necessary and sufficient condition. In addition, extensions to the case of systems with input constraints and uncertainty are also one of the significant topics.
